# Experimental Testing of an Approach to Establishing Combined Toxicity of Ternary Nanoparticle Mixtures

**DOI:** 10.3390/ijms23084356

**Published:** 2022-04-14

**Authors:** Ilzira A. Minigalieva, Vladimir G. Panov, Vladimir B. Gurvich, Larisa I. Privalova, Svetlana V. Klinova, Boris A. Katsnelson

**Affiliations:** 1 Yekaterinburg Medical Research Center for Prophylaxis and Health Protection in Industrial Workers, 620014 Yekaterinburg, Russia; ilzira-minigalieva@yandex.ru (I.A.M.); vpanov@ecko.uran.ru (V.G.P.); gurvich@ymrc.ru (V.B.G.); privalovali@yahoo.com (L.I.P.); klinovasv@ymrc.ru (S.V.K.); 2 Institute of Industrial Ecology, Ural Branch of the Russian Academy of Sciences, 620049 Yekaterinburg, Russia

**Keywords:** ternary nanoparticle mixtures, combined toxicity, mathematical modelling

## Abstract

Our studies of exposure to binary mixtures of nanoparticles (TiO_2_ + SiO_2_; TiO_2_ + Al_2_O_3_ and SiO_2_ + Al_2_O_3_) based on mathematical modelling show that their combined subchronic toxicity can either be of an additive type or deviate from it depending on the outcome, dose ratio, and levels of effect. To characterize the type of toxicity of ternary mixtures of nanoparticles, we successfully tested a previously developed approach for assessing the combined toxicity of metal ions. In this approach, the effects are classified by a null, positive, or negative change in the toxicity of binary nanoparticle mixtures when modeled against the toxicity of the third agent added.

## 1. Introduction

In 2015, we [1] published the results of an experiment in which outbred white rats were subjected to repeated injections of single, binary, and ternary mixtures of Mn, Ni, or Cr salts as water solution at doses that are isoeffective to acute lethal toxicity doses. Control rats were injected with distilled water at the same volume. Judging by more than 30 indices describing the condition of the rat organism, all the chemicals we tested caused mild-to-moderate intoxication. Mathematical modeling showed that, for each binary exposure (manganese plus nickel, manganese plus chromium, or nickel plus chromium), the combined toxicity (similar to that of lead plus cadmium or lead plus fluoride combinations previously described elsewhere [2,3]) was either of a unidirectional additive type, or departed from it (usually towards subadditivity), depending on the type and level of the effect and on the dose.

As for ternary mixtures of toxicants in full dose, we proposed a novel approach to characterizing their effects based on the consideration whether addition of a third agent to the mixture of the other two led to a negative (Class A), positive (Class B), or no change (Class C) in response. We revealed some stable patterns in this classification that reproduced fully or partly when Mn, Ni, or Cr were added one by one as a third component to the combination. Thus, for the absolute majority of health effects, the classification proved to be inherently consistent.

We maintained that the relevance of the proposed classification to health risk analysis and management was as follows. When assessing the total health risk from exposure to three toxicants using the conventional approach based on summation of single risks, it is essential to take into account the probability of under- or overestimation of the result depending on the classification of the effect. Most deleterious Class A effects are more important in terms of the precautionary paradigm, while the effects of Classes B and C, denoting positive and null changes, respectively, may be considered as providing an additional margin of safety rather than justifying less conservative risk management scenarios.

Besides, we found a complicated reciprocal influence of combined metals on their retention in the kidneys, liver, spleen, and brain, which might presumably be one of the possible mechanisms of combined toxicity, but not always the most important one.

Later on, we tested a similar approach for assessing the combined toxicity of metals as nanoparticles.

The master alloy production process is associated occupationally with workspace air pollution by a combination of metal oxide nanoparticles (MeO NPs) arising as by-products of the technology. In multicomponent chemical pollution settings, there are typically two or three air pollutants observed in the highest concentrations and/or posing a major hazard that are given priority. A review of industrial air quality monitoring data showed that the priority air contaminants for the production of master alloys are aluminum, titanium, and silicon, the health effects of which have been described in numerous studies [4,5,6,7,8,9,10,11]. However, the combined three-factor toxicity of their nanoparticles has never been investigated before. The present article does not provide an overview of the toxicity effects and mechanisms of these elements taken separately because they have been described in detail elsewhere [4,5,6,7,8,9,10,11] and also because we reported them previously [12].

## 2. Results and Discussion

As can be seen from Table 1, only eight indices in the subchronic ternary (Al_2_O_3_, SiO_2_ and TiO_2_) exposure group of rats were significantly different from those in control animals.

The experiment also included a group of rats administered half doses of the MeO NP species, mainly for the construction of a mathematical model of combined toxicity using the response surface method. To avoid complicating the table further, we did not include data for this group and just specified that only two out of the seven indices, in which the Al_2_О_3_ + SiO_2_ + ТiO_2_ exposure group was significantly different from the control, ceased to be so different in response to dose reduction, as follows: brain weight decrease and reduced glutathione in blood hemolysate. Further to the above, we can highlight the normalization of several indices in which the shift caused by the combination under consideration in full dose was statistically insignificant but quite obvious, as follows: a decrease in blood serum lactate dehydrogenase and urea, urinary coproporphyrin, and 24-h urine volume.

**Figure 1:**
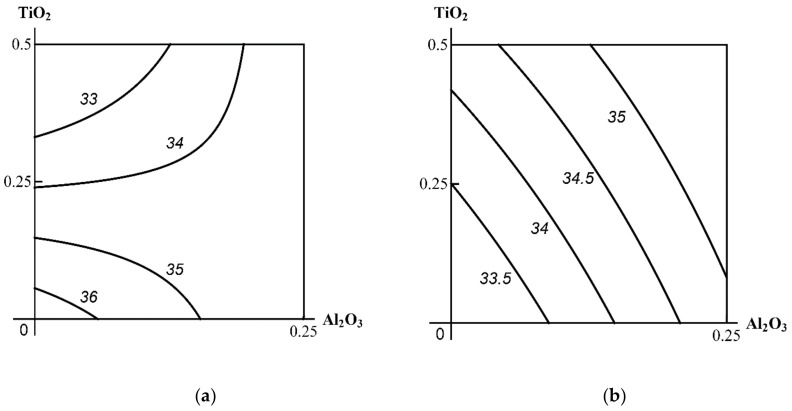
Examples of Class A three-factor toxicity: (**a**) antagonism or contradirectional effect of the Al_2_O_3_ + TiO_2_ combination (for different levels of effect and doses) on serum creatinine in the absence of a third agent changes (for all ratios of effects and doses) into (**b**) additivity in the presence of SiO_2_ NPs. MeO NP doses on the axes are plotted in mg per rat. Numbers on the isoboles show effect values (μmol/L).

**Figure 2:**
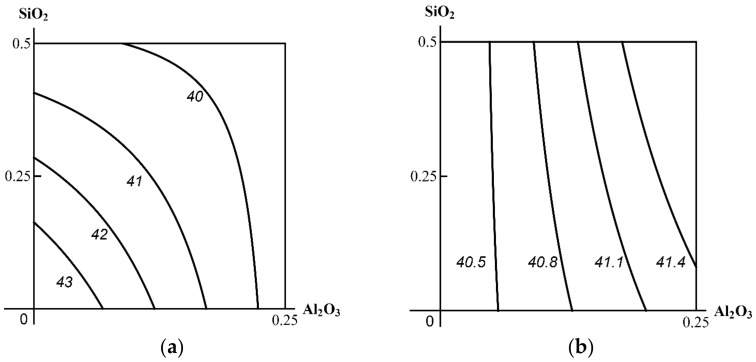
Examples of Class B three-factor toxicity: (**a**) unidirectional antagonism in the effect of SiO_2_ + Al_2_O_3_ nanoparticles on serum albumin changes in the absence of a third agent into (**b**) a single-factor effect of Al_2_O_3_ alone in the presence of TiO_2_ nanoparticles acting simultaneously in the background. MeO NP doses on the axes are plotted in mg per rat. Numbers on the isoboles show effect values (μmol/L).

**Figure 3:**
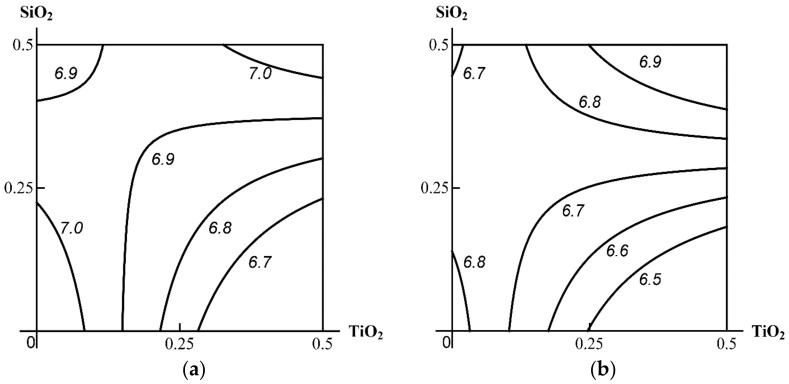
Examples of Class C three-factor toxicity: almost complete coincidence between the isobolograms for the combined effect of SiO_2_ + TiO_2_ nanoparticles on blood serum glucose (**a**) in the absence of and (**b**) in the presence of Al_2_O_3_ NPs. MeO NP doses on the axes are plotted in mg per rat. Numbers on the isoboles show effect values (μmol/L).

Thus, the toxic effect of the ternary combination assessed by shifts in the functional and biochemical indices of the rat organism appeared to be minor, and, moreover, it did not demonstrate any explicit dose dependence. Neither were there any statistically significant differences from the same indices of the three binary exposure groups. A two-stage analysis gave a clearer picture of the combined 3-factor toxicity. Figure 1, Figure 2 and Figure 3 demonstrate the examples pertinent to the Al_2_О_3_ + SiO_2_ + ТiO_2_ nanoparticle exposure.

In general, out of the toxic effects in Table 1 that could be reliably classified (150 in total from all variants of considering one of the toxic agents as a background one), 35% fell into Class A, 43% into Class B, and 22% into Class C. Class A was somewhat prevalent for Al_2_O_3_ as the third factor, while for the other two MeO NPs, it was Class C that prevailed (44%).

**Figure 4:**
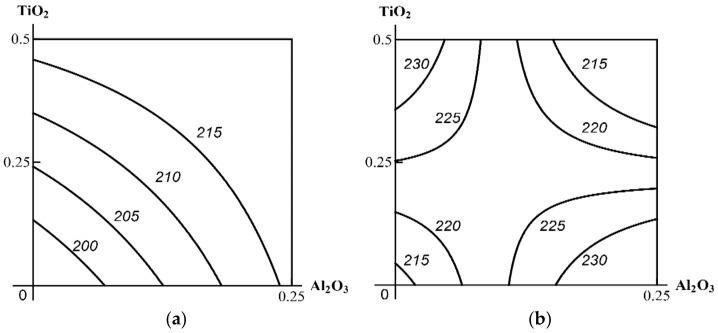
An example of three-factor toxicity of undefined class: the combined effect of Al_2_O_3_ + TiO_2_ nanoparticles on alkaline phosphatase activity in blood serum (**a**) in the absence of a third factor, and (**b**) in the presence of SiO_2_ (see the text for interpretation). MeO NP doses on the axes are plotted in mg per rat. Numbers on the isoboles show effect values (U/L).

At the same time, there are cases noted in modeling the impact of this ternary combination where an effect could not be ambiguously referred to one of the following three classes: in some parts of the range of combined doses, the change of the binary toxicity type in the presence of a third agent may be regarded as more detrimental (Class A), while in others as less detrimental (Class C) to health. A typical example of this kind is illustrated by the isobolograms in Figure 4.

In this case, the effect of Al_2_O_3_ + TiO_2_ on alkaline phosphatase activity in blood serum in the absence of the third agent was additive over the entire range of doses with a certain tendency towards antagonism, whereas in the presence of SiO_2_ NPs, this type of Al_2_O_3_ + TiO_2_ combined toxicity persisted only at the lowest doses of these nanoparticle species (Class C). At the same time, combinations of high doses of any of them with low doses of the other demonstrated different variants of opposite action (Class B), while combinations of high doses of both factors acted unidirectionally in synergism (Class A).

In such complicated cases, it is unlikely to be possible to formalize the determination of the prevailing class of 3-factor combined toxicity, and the choice should be based on expert judgement, taking into account various toxicological and hygienic considerations, but generally proceeding from the precautionary principle. In the above case, for instance, the change of any type of binary toxicity to synergism in some parts of the dose range should prevail in the latter, i.e., predetermine its classification into Class A.

Table 2 shows the morphometric indices of binary combinations’ nephrotoxicity in comparison with the indices for the ternary combination (for full and halved doses of each MeO NP species). It should be noted that the addition of SiO_2_ to the most nephrotoxic combination of Al_2_O_3_ + TiO_2_ enhanced the effect slightly (possible antagonism), while Al_2_O_3_ added to the combination of SiO_2_ + TiO_2_ doubled it to a statistically significant level, though for brush border loss only. Thus, as in the above analysis of functional and biochemical effects, Al_2_O_3_ NPs again seem to play a leading role (even at a relatively low dose) in the combined toxicity of MeO NPs, though in relation to an isolated impact on the kidneys. The association of this effect with exposure to the combination under study is, in itself, confirmed by its obvious dependence on the effective dose of the entire combination.

As rough mathematical estimates show, whereas the sum of increases in brush border loss over the three agents compared to the control value is equal to 3.23 (Al_2_O_3_ = 0.36, TiO_2_ = 2.12, and SiO_2_ = 0.75), the combined action of these factors raises this index to 5.70, which points to a possible synergism. Similar summation of the values for the second effect of nephrotoxicity (desquamation of tubular epithelial cells, %) gives an expected value of 0.87 (Al_2_O_3_ = 0.15, TiO_2_ = 0.42, and SiO_2_ = 0.3) for the sum of three isolated effects and 1.04 for the actual combined effect, also suggesting some synergism.

**Figure 5:**
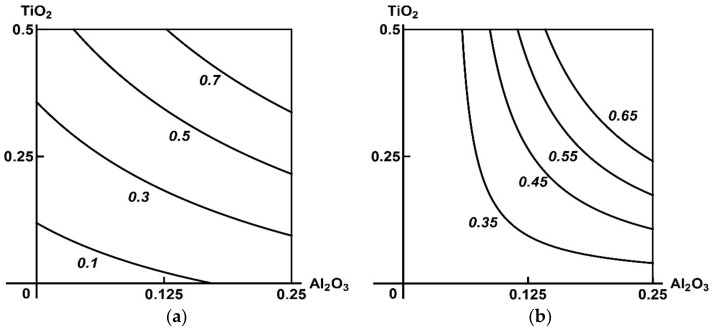
An example of Class A three-factor toxicity. The isobolograms of Al_2_O_3_ + TiO_2_ combined subchronic nephrotoxicity as assessed by desquamation of renal tubular epithelial cells as follows: (**a**) additivity of the unidirectional actions in the absence of a third agent; (**b**) synergism in the presence of SiO_2_ NPs. MeO NP doses on the axes are plotted in mg per rat. Numbers on the isoboles show effect values (in %).

At the same time, the type of binary effect in relation to the morphometric indices of nephrotoxicity under consideration may change more or less significantly under the impact of the third agent towards a higher hazard (Class А), as shown in Figure 5.

**Figure 6:**
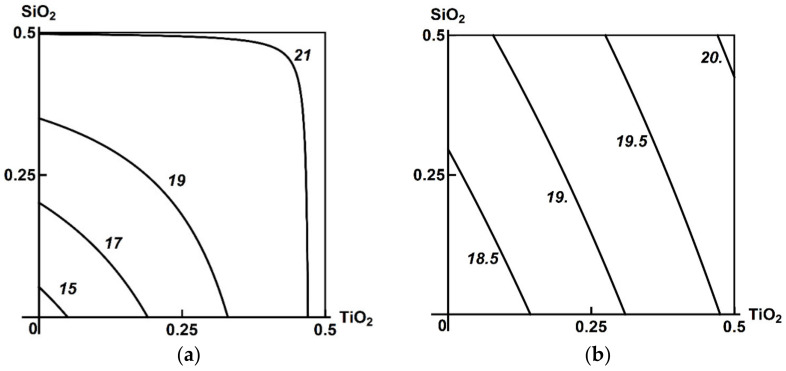
An example of Class A three-factor toxicity. Isobolograms of SiO_2_ + TiO_2_ combined subchronic liver toxicity assessed by the increase in the number of Kupffer cells as follows: (**a**) antagonism of unidirectional actions in the absence of a third agent and (**b**) additivity in the presence of Al_2_O_3_ NPs. MeO NP doses on the axes are plotted in mg per rat. Numbers on the isoboles show effect values.

Comparison of the binary and ternary exposure groups (Table 3) gives an impression of antagonism as the predominant type of combined hepatotoxicity of the MeO NPs under study, which was generally confirmed by RSM modelling. Figure 6 demonstrates that, in this case as well, that the presence of a third component could change the type of combined action of the other two towards more harmful for the organism (Class A).

**Figure 7:**
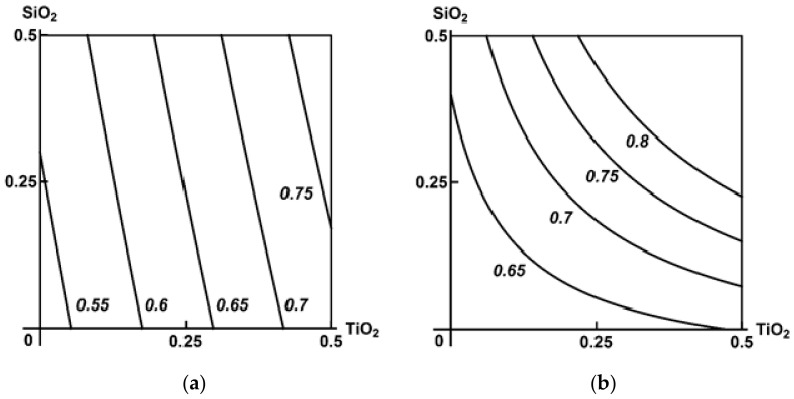
Isoboles for the combined subchronic toxicity of SiO_2_ + TiO_2_ for spleen assessed by the increase in the planimetric white to red pulp ratio as follows: (**a**) additivity of unidirectional actions in the absence of Al_2_O_3_ NP and (**b**) synergism in its presence (an example of Class A three-factor action). MeO NP doses on the axes are plotted in mg per rat; the numbers on the isoboles show ratio values.

Class A was also established when assessing toxicity by the increase in the white to red pulp area ratio of the spleen, which was usually higher when Al_2_O_3_ was considered as the third component of the combination (Figure 7).

A decrease in the dose of the ternary combination was found to halve the hepatotoxic effect judging by just its direct and unambiguously interpreted morphometric index, i.e., the number of prokaryotic cells (Table 3).

A similar dose dependence, although statistically insignificant, is also demonstrated by the impact of the ternary combination on the planimetric white to red pulp area ratio of the spleen, this effect being higher in both dosage groups compared to the effect of any binary combination.

Finally, the same patterns of effect produced by the combination under consideration were revealed in the DNA fragmentation factor as an index reflecting its genotoxicity (Table 4).

The association between this effect and toxic exposure is again evidenced by the dependence of the first on the dose of the second. Indeed, whereas the ternary combination of the nanoparticle species in full dose increased the fragmentation factor 1.6 times against the control value, the combination of half doses increased it 1.2 times only (*p* < 0.05). The genotoxic effects of the three binary combinations were higher (although not always significantly) than the effects of the individual agents in them taken separately, the presence of Al_2_О_3_ in the combination being of greatest importance here as well. Considering again the difference between DNA fragmentation factors for the experimental and control groups as a measure of the genotoxic effect, gives an impression of synergism in this effect of the ternary combination as well as in the effect of binary combinations containing Al_2_О_3_ (especially its combination with TiО_2_). However, in this case as well, the type of combined effect we deal with can only be established in general form based on the results of RSM modeling and the proposed classification.

**Figure 8:**
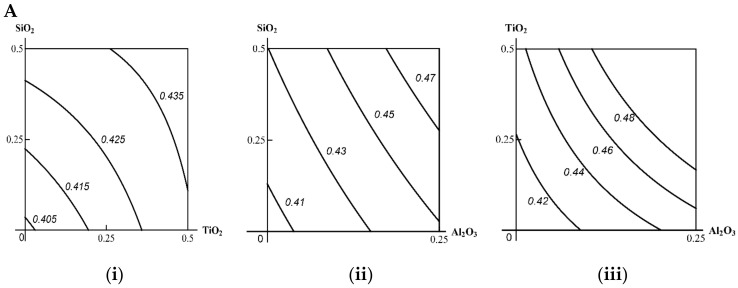

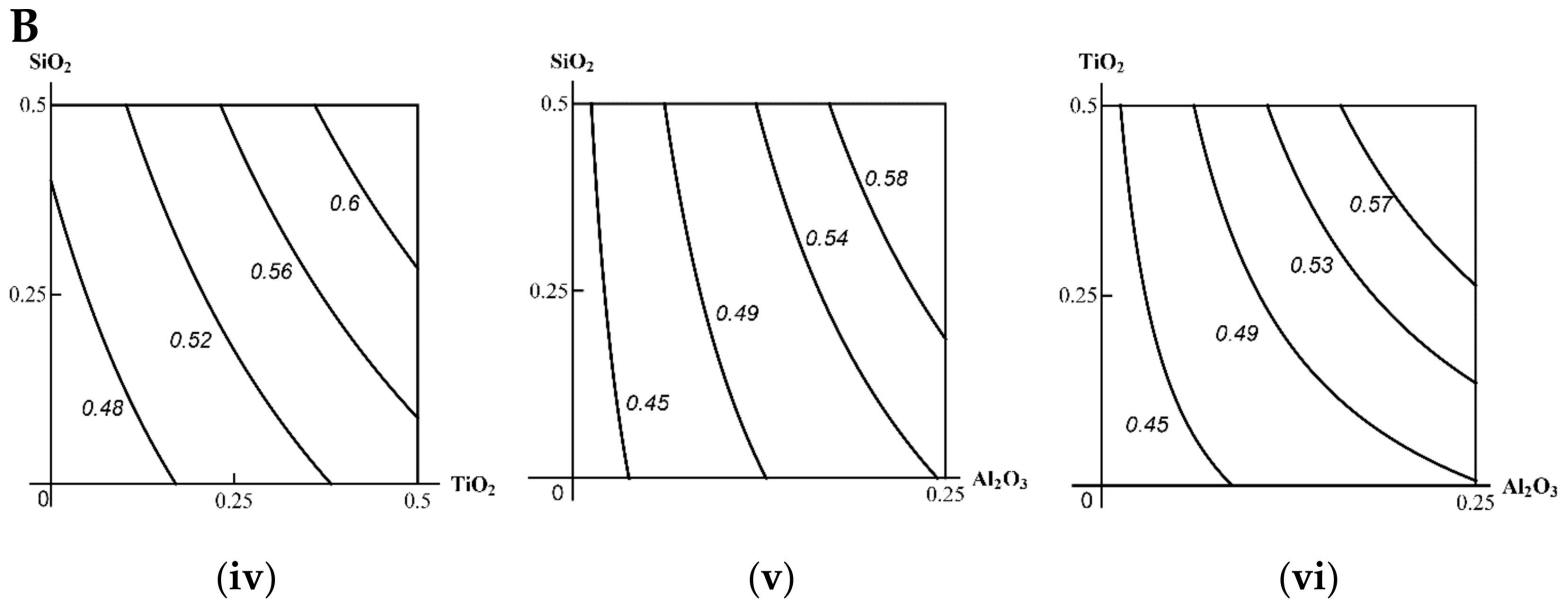
Isobolograms for the binary combined genotoxicity of nanoparticles assessed by the increase in the fragmentation factor of nucleated blood cells as follows: (**A**) in the absence of a third agent under exposure to: (**i**) SiO_2_ + TiO_2_ (antagonism); (**ii**) SiO_2_ + Al_2_O_3_ (additivity); (**iii**) TiO_2_ + Al_2_O_3_ (synergism); (**B**) in the presence of a third agent: (**iv**) Al_2_O_3_ combined with SiO_2_ + TiO_2_ (additivity); (**v**) TiO_2_ combined with SiO_2_ + Al_2_O_3_ (a single-factor effect transforming into additivity); (**vi**) SiO_2_ combined with ТiO_2_ + Al_2_O_3_ (synergism). The axes plot the doses of relevant MeO NPs in mg per rat; the numbers on the isoboles denote the dimensionless value of the fragmentation factor.

As the isoboles in Figure 8 suggest, when Al_2_O_3_ is considered as a background (third) component, the obvious antagonism in the effect of the other two turns into additivity with a tendency towards synergism, i.e., the type of combined action that is more harmful for the organism (Class A). However, if the third component is TiO_2_, then the genotoxicity of SiO_2_ + Al_2_O_3_, which was strictly additive in its absence, approximates a single-factor effect, determined mainly by the dose of Al_2_O_3_, which is less unfavourable (Class B). Finally, SiO_2_ added to ТiO_2_ + Al_2_O_3_ did not change the super-additive type of action of the latter (Class C). Thus, as in the case of functional and biochemical indices of combined toxicity, the classification of the three-factor genotoxicity of Al_2_O_3_ + TiO_2_ + SiO_2_ NPs appears to be ambiguous, depending on which of these factors is considered as the third one. The precautionary principle, which is crucial with respect to genotoxic effects, suggests that it would be correct to refer this ternary combination to the most unfavorable Class A, and so much more so that the latter is determined by the factor of Al_2_O_3_, which both by itself and in binary combinations, proved to be the most hazardous.

## 3. Materials and Methods

### 3.1. Nanoparticles

Airborne particles were sampled on polycarbonate filters in the workspace of the aluminum titanium master alloy smelting shop at VSMPO-AVISMA Corporation, Verkhnyaya Salda, Sverdlovsk Region, Russia, and analyzed for elemental composition by SEM-energy-dispersive spectroscopy using the Carl Zeiss AURIGA^®^ CrossBeam^®^ Workstation (Carl Zeiss NTS, Oberkochen, Germany). Table 5 demonstrates the prevalence of the following three elements in the average composition of the samples: titanium (17.5%); aluminum (14.8%); silicon (12.0%), represented by oxides of these metals.

**Figure 9:**
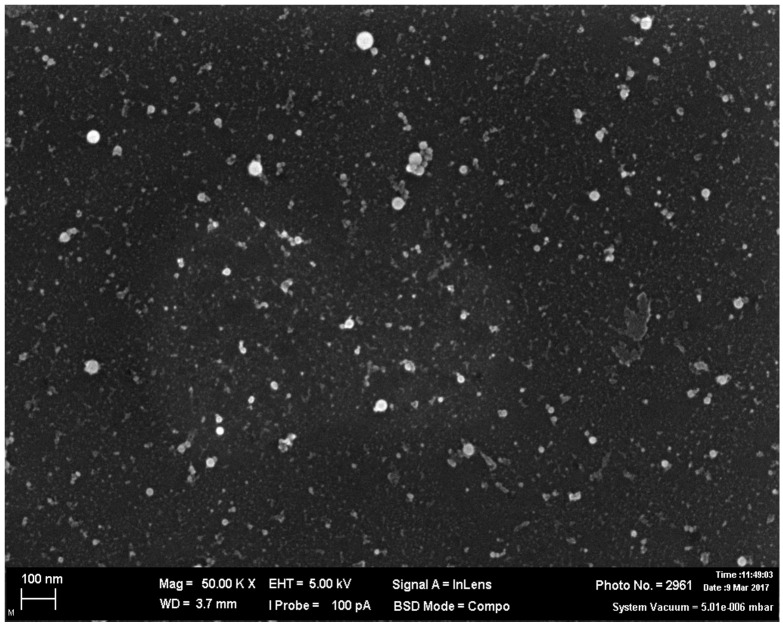
A SEM image of Al_2_O_3_ particles at 50,000× magnification.

**Figure 10:**
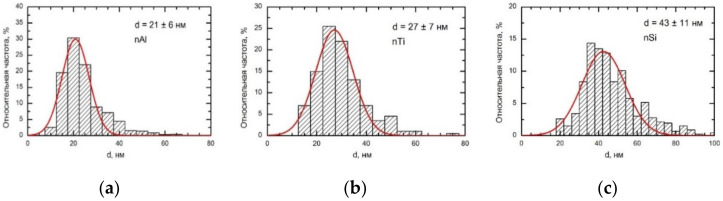
Size distribution of the nanoparticles used in the experiment ((**a**)—Al_2_O_3_; (**b**)—TiO_2_; (**c**)—SiO_2_).

Based on the above data, aqueous suspensions of TiO_2_, SiO_2_, and Al_2_O_3_ nanoparticles were specially prepared for the experiment by laser ablation of the surface of a superpurity metal plate under a layer of deionized water. The particles had spherical or near-spherical shape (see Figure 9 showing a scanning electron microscopy (SEM) image of Al_2_O_3_ particles as an example) and a symmetrical size distribution (Figure 10) with the mean (±s.d.) diameters of 27 ± 7 nm for TiO_2_, 43 ± 11 nm for SiO_2_, and 21 ± 6 nm for Al_2_O_3_.

### 3.2. Animal and Intoxication Model

Subchronic exposure to metal oxide nanoparticles (MeO NPs) was modelled by repeated intraperitoneal injections of corresponding suspensions into four-month-old outbred rats of our own breeding with an initial body mass of 250–350 g. Six groups of 12 male rats each were exposed thrice a week for six weeks. A single dose of SiO_2_ or TiO_2_ was equal to 0.5 mg per milliliter of suspension, and that of Al_2_O_3_ to 0.25 mg/mL as it was impossible to obtain a more concentrated suspension of the latter. Additionally, we used one group under ternary exposure in which these nanoparticles were injected at two times lower doses each. In Table 2 this exposure is designated as “Al_2_O_3_ + SiO_2_ + TiO_2_ (half dose)”. As only morphometrically assessed responses of kidneys proved dependent on such dose decrease, we have not included functional responses of this additional group in Table 1.

The animals were kept in a specially organized compartment of the vivarium in compliance with the International Guiding Principles for Biomedical Research Involving Animals developed by the Council for International Organizations of Medical Sciences, and the International Council for Laboratory Animal Science (2012). The experiment was approved by the Local Bioethics Committee of the Yekaterinburg Medical Research Center for Prophylaxis and Health Protection in Industrial Workers, Yekaterinburg, Russia.

### 3.3. Postexposure Indices of the Rat Organism

More than 50 physiological, cytological, and biochemical indices of control and experimental animals were assessed following the six-week exposure period, during which the health status of the rats was monitored regularly and seven body weight measurements were taken, the last one immediately preceding euthanasia.

To study the function of the central nervous system, we applied the summation threshold index most frequently used in experimental toxicology [13]. The higher nervous activity of the animals was characterized by their head dipping behavior, i.e., the total number of head dips into the holes over a 3-min period [14,15].

The rats were quickly sacrificed by decapitation under raush narcosis and their blood was collected to analyse hTmoglobin, red blood cell count, the proportion of reticulocytes, white blood cell count, and succinate dehydrogenase (SDH)|activity in blood lymphocytes. The levels of hTmoglobin and red blood cell count were measured using a Mythic 18 analyzer with appropriate diagnostic kits; the white blood cell count was performed on stained blood smears. SDH activity was assessed cytochemically using paranitroviolet tetrazolium and expressed as the number of formazan granules in 50 cells [16].

An important additional functional indicator of erythropoiesis is an elevated count of reticulocytes, which are immature red blood cells developing after the loss of nuclei by normoblasts. A characteristic feature of reticulocytes is the presence of cytoplasmic granular and filamentous substances in their cytoplasm detected by supravital staining (i.e., without prior fixation) with brilliant cresyl blue. The number of reticulocytes was counted per 1000 erythrocytes [17].

The liver function was assessed by the indices of total protein, albumin to globulin ratio, and alanine aminotransferase and aspartate aminotransferase activity [17].

Coproporphyrin and delta-aminolevulinic acid levels in urine were used to estimate porphyrin metabolism [17].

We also measured the serum malondialdehyde and catalase content to assess lipid peroxidation and antioxidant mechanisms in the organism [17].

Along with the above techniques, we evaluated the genotoxicity of this combination of substances. One of the most commonly used methods of genotoxicity assessment is the amplified fragment length polymorphism assay. It allows detecting DNA fragmentation, i.e., estimating quantitatively the degree of genotoxicity of damaging agents used. The leukocyte fraction is isolated from the obtained whole blood samples using one-step ficoll-verografin density gradient separation. The samples are then grouped in accordance with the results. DNA is isolated by acid phenol-chloroform extraction. The polymerase chain reaction is carried out in LaMMT modification increasing the PCR sensitivity by 8–10 times. To run the reaction, use is made of specific tritium-labelled primers and nucleotides (dCTP, dATP, and methyl-dTTP). The resulting PCR amplification product is separated by horizontal agarose gel electrophoresis in TAE buffer at 100 V for 15 min.

Following the electrophoresis, the gel plates are divided into tracks, each of which is cut into 5-mm-long sections. The resulting gel fragments are placed in vials containing 3.0 mL of absolute isopropanol. The vials are heated to 80 °С for two hours. After extraction of the labelled amplified fragments from the gel, 6.0 mL of a simple toluene scintillator is added to the vials followed by liquid scintillation counting using an automated Beta-2 counter. The ratio of radioactive activity in the “tail” and “head” of the preparation is a quantitative measure of DNA fragmentation (i.e., a fragmentation factor).

Differences between the group averages were processed using the Student’s *t*-test in Microsoft Excel. These differences were considered statistically significant if the probability of a random difference did not exceed 5% (*p* < 0.05).

### 3.4. Mathematical Description of Combined Toxicity

The first stage of the analysis involved classification of all quantitative indices of the toxic effects under study by type of combined toxicity of each combination of two factors (TiO_2_ + SiO_2_, TiO_2_ + Al_2_O_3_, or SiO_2_ + Al_2_O_3_) in the absence or presence of a third one. At this stage, the response of a certain index to the combined (binary) exposure was mathematically described using isobolograms constructed from the following regression model:

Y = b_0_ + b_1_ ∗ x_1_ + b_2_ ∗ x_2_ + b_12_ ∗ x_1_ ∗ x_2_, (1)

which was discussed and illustrated with examples in detail elsewhere [2,3].

In fact, the Equation (1) is the analytical model of the response surface. The virtual “sectioning” of this surface on different levels corresponding to different values of the outcome Y or of the doses x, provides a family of Loewe isoboles that may have the same or a different form and/or different slopes, and thus render the interpretation of binary combined toxicity types both easy and illustrative.

As a principle of toxicological characterization of a ternary mixture of toxicants, we applied the classification of changes from one type of combined effect produced by two toxicants to another following the addition of a third agent, meaning (A) deterioration, (B) improvement, or (C) no change. In accordance with this classification, Class A, representing a negative change, includes indices undergoing one of the following transitions:

- No statistically significant effect of both toxicants taken together or separately was observed initially in the absence of the third agent but an adverse effect was revealed following the addition of the latter;
- The initial situation was characterized by antagonism under a unidirectional effect of the toxicants while the final state was characterized by homogeneous additivity and even synergism (in at least one range of doses and/or levels of response);
- The initial situation was characterized by homogeneous additivity under a unidirectional effect of the agents while the endpoint was characterized by synergism (in at least one range of doses and/or levels of response).

Similarly, Class B that shows a positive change following the addition of the third agent describes the following opposite transitions:

- From any type to the type of no effect of each of the toxicants along and in combination;
- From synergism (detected in at least one range of doses and/or levels of response) to uniform additivity and, even more so, to antagonism in at least one range of doses and/or levels of response under the unidirectional effect of the toxicants;
- From uniform additivity to subadditivity of a unidirectional effect or counter-directional effect in at least one range of doses and/or response levels.

Finally, Class C represents no change from one type of combined toxicity to another, as well as changes that do not modify the overall qualitative picture of the combined effect (e.g., transition from subadditivity of a unidirectional effect to the formal additivity of the counter-directional effect is interpreted as maintenance of antagonism as a generalized class of a relatively favourable type of combined toxicity).

## 4. Conclusions

To assess the combined effect of metal oxide nanoparticles, we applied the classification of combined three-factor toxicity based on a two-stage approach, enabling changes in the type of binary toxicity following the addition of a third agent to be identified. This assessment was performed by mathematical modeling of combined toxicity types, which showed that the outcomes of exposure to a ternary mixture can be more or less noxious (Classes A and B, respectively) or remain unchanged (Class C) following the addition of a third component. When assessing the cumulative health risk from exposure to a ternary mixture of toxicants based on the generally accepted approach of single factor risk summation, it is essential to take into consideration that the result of such an assessment may be either underestimated (if the toxicity effects fall into Class A) or somewhat overestimated (if they fall within Class B). The first option is more important from the perspective of the precautionary principle, while the second one should be considered only as an additional margin of safety.

## Figures and Tables

**Table 1:** Some functional indices of the rat organism following 18 intraperitoneal injections of MeO NPs in binary and ternary combinations administered during six weeks ($\bar{X}$ ± Sx).

Indices	Control	Al_2_O_3_ + TiO_2_	Al_2_O_3_ + SiO_2_	TiO_2_ + SiO_2_	Al_2_O_3_ + SiO_2_ + TiO_2_
		Group 1	Group 2	Group 3	Group 4
Initial body weight, g	292.27 ± 5.020	287.08 ± 7.20	290.83 ± 7.50	291.67 ± 7.40	290.00 ± 5.50
Final body weight, g	332.27 ± 7.90	320.00 ± 3.50	322.50 ± 5.20	324.17 ± 7.70	334.55 ± 8.10
Weight gain, %	15.13 ± 1.80	13.41 ± 2.00	14.70 ± 1.80	12.58 ± 2.00	15.40 ± 2.00
Number of head dips into holes in 3 min	4.73 ± 0.94	5.08 ± 1.02	3.00 ± 0.59	4.42 ± 0.67	3.27 ± 0.78
Number of squares crossed in 3 min	8.18 ± 1.25	7.58 ± 1.17	5.00 ± 0.55 *	7.58 ± 1.19	6.50 ± 1.09
Summation threshold index, s	14.27 ± 1.29	15.35 ± 0.85	14.88 ± 1.08	13.39 ± 0.86	14.41 ± 0.97
Left kidney weight, g/100 g body weight	0.30 ± 0.01	0.30 ± 0.01	0.29 ± 0.01	0.30 ± 0.01	0.28 ± 0.01
Right kidney weight, g/100 g body weight	0.31 ± 0.01	0.30 ± 0.01	0.30 ± 0.01	0.30 ± 0.01	0.29 ± 0.01
Liver weight, g/100 g body weight	3.08 ± 0.13	3.22 ± 0.18	3.22 ± 0.16	3.19 ± 0.13	3.09 ± 0.16
Spleen weight, g/100 g body weight	0.17 ± 0.01	0.18 ± 0.01	0.17 ± 0.01	0.18 ± 0.01	0.18 ± 0.01
Left testis weight, g/100 g body weight	0.55 ± 0.02	0.55 ± 0.01	0.55 ± 0.01	0.53 ± 0.01	0.53 ± 0.02
Right testis weight, g/100 g body weight	0.55 ± 0.02	0.54 ± 0.01	0.55 ± 0.01	0.53 ± 0.01	0.53 ± 0.02
Brain weight, g/100 g body weight	0.62 ± 0.01	0.61 ± 0.01	0.61 ± 0.01	0.61 ± 0.01	0.59 ± 0.01 *
Hemoglobin, g/L	158.89 ± 1.16	147.33 ± 2.8 *	146.00 ± 1.5 1 *	151.25 ± 2.45 *	147.75 ± 2.28 *
Erythrocytes, 10^12^/L	7.93 ± 0.16	7.61 ± 0.24	7.48 ± 0.11 *	7.52 ± 0.10 *	7.83 ± 0.17
Mean corpuscular volume, μm^3^	54.69 ± 0.86	52.17 ± 0.67 *	52.36 ± 0.57 *	54.94 ± 0.344	51.73 ± 0.93 *
Reticulocytes, ‰	13.63 ± 1.65	29.90 ± 1.28 *	25.86 ± 1.61 *	31.67 ± 2.73 *	15.50 ± 1.41 ^1^^,^^2,^^3^
Hematocrit, %	21.54 ± 0.21	20.03 ± 0.69 *	19.59 ± 0.26 *	20.65 ± 0.28 *	20.20 ± 0.30 *
Thrombocytes, 103/μL	847.25 ± 25.41	831.75 ± 54.09	926.57 ± 7.89	880.50 ± 34.53	882.25 ± 36.87
Thrombocrit, %	0.23 ± 0.02	0.24 ± 0.02	0.27 ± 0.01	0.25 ± 0.01	0.26 ± 0.01
Leucocytes, 103/μL	7.20 ± 0.37	9.10 ± 1.03	9.40 ± 0.83 *	7.85 ± 0.67	7.78 ± 0.66
Eosinophils, %	2.20 ± 0.29	3.13 ± 0.484	3.57 ± 0.87	2.13 ± 0.40	3.00 ± 0.42
Segmented neutrophils, %	19.50 ± 0.64	20.000.82	20.43 ± 1.91	20.50 ± 0.98	20.75 ± 1.05
Band neutrophils, %	1.50 ± 0.17	1.00 ± 0.00 *	2.29 ± 0.29 *^,^^1^	1.38 ± 0.18 ^2^	1.63 ± 0.26
Monocytes, %	6.20 ± 0.39	6.75 ± 0.49	6.86 ± 0.34	6.38 ± 0.42	6.25 ± 0.45
Lymphocytes, %	70.60 ± 0.95	69.13 ± 0.91	66.71 ± 2.60	69.63 ± 1.13	68.50 ± 1.24
Succinate dehydrogenase (SDH) in blood lymphocytes, number of formazan granules	589.45 ± 16.55	562.67 ± 15.74	551.55 ± 20.54	530.42 ± 16.03 *	561.64 ± 15.99
Gamma-glutamyltransferase (GGT), IU/L	2.26 ± 0.69	2.48 ± 0.45	3.98 ± 0.99	0.74 ± 0.30 ^2^	1.10 ± 0.66 ^2^
Glucose, mol/L	7.09 ± 0.26	6.10 ± 0.30 *	6.64 ± 0.18	7.04 ± 0.29	7.08 ± 0.18 ^1^
Serum ceruloplasmin, mg/%	33.14 ± 0.13	44.06 ± 1.53 *	46.22 ± 2.35 *	42.88 ± 1.44 *	42.61 ± 1.88 *
Serum malondialdehyde (MDA), μmol/L	3.51 ± 0.49	3.56 ± 0.48	5.10 ± 0.37 *	4.57 ± 0.19 *	4.28 ± 0.29
Serum catalase, μmol/L	1.34 ± 0.25	1.31 ± 0.22	1.18 ± 0.24	0.65 ± 0.12 *	1.10 ± 0.21
Reduced glutathione in whole blood, μmol/L	26.82 ± 1.19	26.20 ± 0.87	28.44 ± 1.47	26.00 ± 1.39 ^2^	22.55 ± 1.41 *^,^^1,^^2^
Plasma sulfhydryl groups, mmol/L	37.33 ± 7.38	36.93 ± 6.04	43.21 ± 6.99	42.72 ± 6.90	40.20 ± 6.66
Serum total protein, g/L	80.47 ± 1.42	80.49 ± 2.01	78.20 ± 1.33	78.93 ± 2.15	79.91 ± 1.82
Serum albumin, g/L	44.34 ± 0.61	41.31 ± 1.05 *	39.58 ± 0.67 *	40.18 ± 1.24 *	41.91 ± 0.88 *
Serum globulin, g/L	36.13 ± 1.22	39.18 ± 1.54	38.63 ± 1.06	38.75 ± 1.53	38.00 ± 1.40
Atherogenic index of plasma	1.24 ± 0.04	1.06 ± 0.05 *	1.03 ± 0.03 *	1.05 ± 0.05 *	1.11 ± 0.04 *
Serum AST level, U/L	218.44 ± 17.65	264.61 ± 25.72	236.81 ± 17.78	187.39 ± 5.07 ^2^	213.91 ± 17.83
Serum ALT level, U/L	70.82 ± 3.24	66.46 ± 4.41	66.50 ± 1.66	63.94 ± 3.32	66.75 ± 3.55
AST/ALT ratio	3.12 ± 0.24	3.76 ± 0.39	3.55 ± 0.23	3.01 ± 0.23	3.22 ± 0.25
Alkaline phosphatase, U/L	193.64 ± 13.08	222.55 ± 13.71	240.48 ± 21.89 *	236.53 ± 10.62 *	200.30 ± 12.154
Serum creatinine, μmol/L	36.33 ± 1.46	34.46 ± 1.71	34.50 ± 1.49	33.89 ± 1.352	35.39 ± 1.03
Serum bilirubin, μmol/L	1.14 ± 0.13	1.31 ± 0.13	1.09 ± 0.14	0.98 ± 0.14	1.10 ± 0.16
Serum Ca^2+^ concentration, mol/L	2.61 ± 0.03	2.56 ± 0.04	2.57 ± 0.03	2.52 ± 0.05	2.58 ± 0.02
Serum follicle-stimulating hormone, U/L	0.14 ± 0.02	0.13 ± 0.01	0.11 ± 0.01	0.11 ± 0.01	0.14 ± 0.01
Serum luteinizing hormone, U/L	0.13 ± 0.02	0.94 ± 0.58	0.35 ± 0.21	0.77 ± 0.63	0.12 ± 0.01
Serum prolactin, U/L	7.85 ± 0.62	15.71 ± 4.51	8.22 ± 0.82	14.73 ± 5.25	6.84 ± 0.35
Serum testosterone, nmol/L	8.20 ± 3.40	9.77 ± 2.10	8.45 ± 4.01	16.28 ± 4.27	13.34 ± 8.17
Serum lactate dehydrogenase, U/L	1904.10 ± 296.03	2208.25 ± 290.47	2119.50 ± 305.84	1628.50 ± 149.27	1709.88 ± 246.58
Serum uric acid, μmol/L	120.50 ± 10.86	121.00 ± 11.99	122.00 ± 9.19	115.75 ± 8.69	123.63 ± 9.61
Serum urea, mmol/L	4.44 ± 0.34	3.59 ± 0.36	3.71 ± 0.27	3.73 ± 0.37	3.35 ± 0.42
24-h urine volume, mL	29.67 ± 4.36	33.00 ± 2.50	24.86 ± 2.20	31.71 ± 5.64	26.86 ± 4.14
Urinary coproporphyrin, nmol/L	162.42 ± 31.78	135.37 ± 62.57	111.83 ± 39.84	155.14 ± 31.38	76.11 ± 24.08
Daily coproporphyrin, μmol	8.14 ± 3.60	4.63 ± 2.23	5.42 ± 2.46	5.91 ± 2.02	4.34 ± 2.23
Delta-ALA in urine, μg/mL	14.11 ± 3.52	13.87 ± 4.37	9.90 ± 3.98	12.61 ± 2.99	13.48 ± 5.22
Urinary creatinine, mmol/L	1.57 ± 0.11	1.54 ± 0.14	1.92 ± 0.13	1.85 ± 0.17	2.03 ± 0.19 ^1^
Creatinine clearance	1.40 ± 0.15	1.50 ± 0.23	1.38 ± 0.08	1.60 ± 0.19	1.49 ± 0.19
Urinary total protein, g/L	190.43 ± 9.63	196.13 ± 20.43	193.36 ± 20.67	211.45 ± 37.18	233.13 ± 30.83
Urine pH	7.17 ± 0.17	6.79 ± 0.15	7.00 ± 0.29	6.93 ± 0.17	6.93 ± 0.37
Urea in urine, mmol/L	229.30 ± 16.00	211.22 ± 17.474	262.08 ± 19.23	238.15 ± 24.71	289.74 ± 28.72
Uric acid in urine, μmol/L	234.00 ± 38.97	204.29 ± 78.30	201.86 ± 64.52	213.00 ± 51.37	216.00 ± 52.97

Notes: * *p* < 0.05 compared to the control index; superscripted numbers are values that are statistically different from the indices in the groups designated by a corresponding number (based on the Student’s *t*-test).

**Table 2:** Morphometric indices of damage to the epithelium of proximal convoluted tubules in rat kidneys following subchronic exposure to binary and ternary mixtures of Al_2_O_3_, TiO_2_, and SiO_2_ nanoparticles ($\bar{X}$ ± Sx).

Indices	Control	Al_2_O_3_ + TiO_2_	Al_2_O_3_ + SiO_2_	TiO_2_ + SiO_2_	Al_2_O_3_ + SiO_2_+ TiO_2_ (Half Dose)	Al_2_O_3_ + SiO_2_+ TiO_2_ (Full Dose)
Brush border loss, %	1.49 ± 0.56	6.45 ± 1.07 *	4.23 ± 0.80 *	3.64 ± 0.70 *#	3.06 ± 0.84 #	7.19 ± 1.47 *
Epithelial desquamation, %	0.00 ± 0.00	0.97 ± 0.48 *	0.29 ± 0.17	0.14 ± 0.14	0.66 ± 0.47	1.04 ± 0.39 *

Notes: * statistically significant difference from the control value; # from the value of the group exposed to a full-dose ternary mixture (*p* < 0.05 by the Student’s *t*-test).

**Table 3:** Morphometric indices of rat liver and spleen following subchronic exposure to binary and ternary mixtures of Al_2_O_3_, TiO_2_ and SiO_2_ ($\bar{X}$ ± Sx).

Indices	Control	Al_2_O_3_ + TiO_2_	Al_2_O_3_ + SiO_2_	TiO_2_ + SiO_2_	Al_2_O_3_ + SiO_2_+ TiO_2_ (Half Dose)	Al_2_O_3_ + SiO_2_+ TiO_2_ (Full Dose)
*Liver*						
Prokaryotic hepatocytes per 100 liver cells	10.30 ± 1.09	29.45 ±1.47 *	41.88 ± 1.72 *#	41.90 ± 1.48 *#	16.92± 0.81 *#	31.85 ± 1.74 *
Binuclear hepatocytes per 100 liver cells	6.65 ± 0.83	5.13 ± 0.46	3.13 ± 0.3 7 *	3.75 ± 0.52 *	5.00 ± 0.33 *	4.05 ± 0.78 *
Kupffer cells per 100 liver cells	14.28 ± 0.45	19.58 ± 0.60 *	18.80 ± 0.72 *	21.05 ± 0.53 *	20.58 ± 0.48 *	20.08 ± 0.75 *
*Spleen*						
Planimetric ratio of white to red pulp	0.499 ± 0.034	0.624 ± 0.048 *#@	0.632 ± 0.040 *#@	0.753 ± 0.046 *#@	0.916 ± 0.057	0.950 ± 0.053

Notes: * statistically significant difference from the control value; # from the value of the group exposed to a full-dose ternary mixture and @ to a half dose ternary mixtures (*p* < 0.05 by the Student’s *t*-test).

**Table 4:** An increase in the genomic DNA fragmentation factor of nucleated blood cells in rats following 18 intraperitoneal injections (during 6 weeks) of suspensions of various binary and ternary combinations of the MeO NPs as established AFLP assay ($\bar{X}$ ± Sx).

Parameter	Control	Al_2_O_3_ + TiO_2_	Al_2_O_3_ + SiO_2_	TiO_2_ + SiO_2_	Al_2_O_3_ + SiO_2_+ TiO_2_ (Half dose)	Al_2_O_3_ + SiO_2_+ TiO_2_ (Full Dose)
Fragmentation factor	0.4023 + 0.0064	0.5416 + 0.0046 *	0.4872 + 0.0041 *	0.4391 + 0.0061 *	0.4849 + 0.0068 *	0.6430 + 0.0189 *

Notes: * statistically significant difference from the control value; statistically significant differences between the groups administered the ternary mixture of MeO NPs in half and full doses (*p* < 0.05 by the Student’s *t*-test).

**Table 5:** The averaged elemental composition of the aerosol particles sampled in the workspace air of the aluminum titanium master alloy shop (in % to the sum of elements minus carbon and oxygen contained in the filter).

Elements	Percent Content
Al	14.8
As	0.1
Ca	8.2
Cl	5.6
Cr	2.5
Cu	0.1
F	1.1
Fe	2.9
K	3.8
Mg	11.4
Na	6.7
Pb	4.0
S	3.6
Si	12.0
Sn	0.5
Ti	17.5
Zn	5.2
Total	100

## Data Availability

The data presented in this study are available on request from the corresponding author.
